# Coxarthritis as the Presenting Symptom of Gaucher Disease Type 1

**DOI:** 10.1155/2011/361279

**Published:** 2011-03-30

**Authors:** Giacomo Brisca, Maja Di Rocco, Paolo Picco, Maria Beatrice Damasio, Alberto Martini

**Affiliations:** ^1^2nd Division of Paediatrics, G. Gaslini Institute, Largo Gaslini 5, 16129 Genova, Italy; ^2^Department of Diagnostic Imaging, G. Gaslini Institute, Largo Gaslini 5, 16129 Genova, Italy; ^3^2nd Division of Paediatrics, G. Gaslini Institute, University of Genoa, Largo Gaslini 5, 16129 Genova, Italy

## Abstract

Gaucher disease (GD) type 1 is the most common lysosomal storage disorder due to beta glucocerebrosidase deficiency leading to an abnormal accumulation of its substrate, glucocerebroside, in the mononuclear phagocyte system. The disease presentation is usually characterized by signs and symptoms related to hypersplenism, such as splenomegaly, anaemia, thrombocytopenia and leucopenia. Skeletal disease may occur later for the infiltration of bone marrow by macrophages infiltration and bone resorption: bone involvement may be heterogeneously manifested by symptoms ranging from bone crisis to avascular necrosis, osteoporosis and defect in remodeling of long bones. Herein, we report a patient in whom the osteoarticular involvement has been the only symptom of the disease stressing that this unusual presentation of GD has prompted a wide differential diagnosis with more common forms of coxitis.

## 1. Case Report 

A 9-year-old girl, born from healthy and unrelated parents, was admitted because of the abrupt occurrence of hip pain and limping. 

The patient's medical history was not significant: namely, neither previous trauma or infections have been reported. At admission, she was extremely suffering, pale, and sweaty, albeit apyrexial and normotensive. 

Her right hip joint was painful and passive, and active motion of the joint was completely restricted; walking was impossible so the patient was using a wheel-chair. On physical examination no other findings were present. 

Standard X-ray of the coxofemoral joints led to rule out major traumatic lesions and bone profile abnormalities. 

Ultrasound evaluation showed the presence of intra-articular fluid effusion at right hip and synovial thickening. 

Acute phase reactants were slightly increased: erythrocyte sedimentation rate was 62 mm/1 h and C-reactive-protein level 1.24 mg/dL (normal values < 0,46 mg/dL). Total and differential white cell count and haemoglobin level were within the normal range; platelets count was 156 × 103 (normal values >150 × 103). 

Upon this basis the diagnosis of acute coxitis has been considered. Indirect serodiagnosis for the common pathogens causing reactive arthritis (i.e., Yersinia, Shigella, Salmonella, Campylobacter jejuni, Parvovirus B19, Echovirus, Coxsackievirus, CMV, EBV, mycoplasma) as well as throat culture and streptococcal antibody titers were negative. Mantoux tuberculin reaction was also negative. 

Antinuclear autoantibodies, rheumatoid factor, and HLA B 27 haplotype were negative too. 

The clinical picture was characterized by the presence of intense right hip pain unresponsive to non steroid anti-inflammatory drugs: it was therefore started treatment with analgesics and opioids such as tramadol administered by elastomeric device with the guidance of a consultant anaesthesiologist which seemed to be partially effective. 

The most common diseases that cause severe hip pain were considered: in particular to rule out hematological or vascular diseases which can cause bone crisis, further investigations were performed. Chest X-ray was normal; abdominal ultrasonography showed no pathological findings except for a slight spleen enlargement. Peripheral smear and hemoglobin electrophoresis were normal too. 

**Figure 1:**
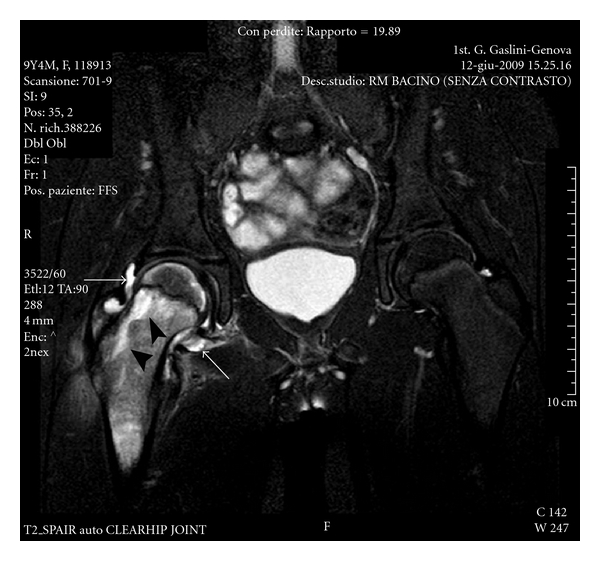
Coronal TSE T2 sequence (TR 2700/TE 70) with fat saturation. Presence of joint effusion in the right coxofemoral joint (arrow) with evidence of marked bone marrow hyperintensity of the femoral head and neck related to bone marrow oedema (arrowhead).

**Figure 2:**
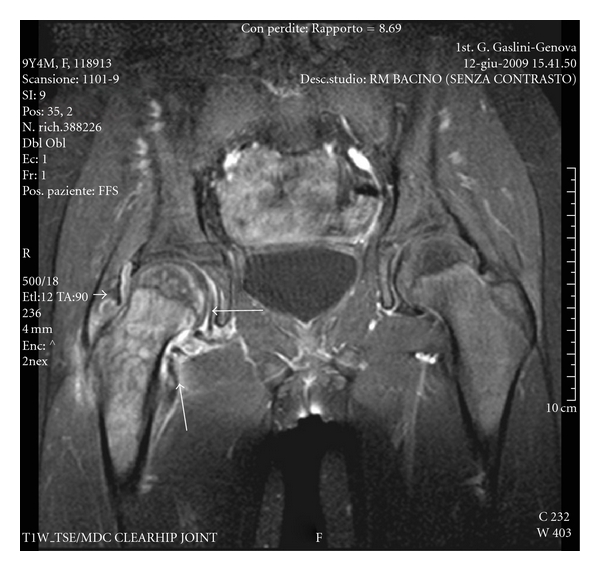
Coronal TSE T1 fat sat (TR 500/TE 18) with fat saturation acquired after MdC injection (Gd-DOTA). Evidence of diffuse articular synovial enhancement (arrows) of the right hip. Inhomogeneous CE is also evident in the femoral head and neck, related to bone marrow oedema, and in the periarticular soft tissues.

The MRI of the hip showed the presence of an effusion in the right coxofemoral joint (Figure 1, arrow) and a marked bone marrow hyperintensity of the femoral head and neck (Figure 1, arrowhead). Gadolinium injection (Gd-DOTA) showed diffuse synovial enhancement and an inhomogeneous contrast enhancement in the femoral head and neck suggestive for bone edema and in the juxtaarticular soft tissues (Figure 2). 

After excluding other possible causes of acute pain/inflammation of the hip joint (e.g., osteomyelitis, septic arthritis, sickle cell disease, haemato-oncologic disorders) the coexistence of an extremely painful hip requiring narcotics, a mild splenomegaly and MRI features resembling massive bone edema, suggested a diagnosis of acute bone crisis in GD. 

The diagnosis was confirmed by a markedly reduced beta glucocerebrosidase enzyme activity in patient's leukocytes: genetic analysis of GBA revealed the presence of compound heterozygosity [c1226A > G(p.N370S)]+[RecNcil]. 

After enzymatic replacement therapy (imiglucerase) the patient did develop neither recurrence of bone disease nor other clinical manifestations related to GD.

## 2. Discussion 

GD is a lysosomal storage disorder caused by deficiency of the enzyme beta glucocerebrosidase, leading to accumulation of glucocerebroside within tissue macrophages in multiple organs. Type 1, non-neuronopathic, is the most common form whilst types II and III are neuronopathic subtypes, more rare, with acute and subacute onset, respectively. 

Although GD has typically been described as an adult disorder affecting mainly Ashkenazi Jews, 49% of GD type I cases are diagnosed before 10 years of age and an additional 17% between 11 and 20 years of age [1]. 

Hepatosplenomegaly, anemia, leukocytopenia, thrombo-cytopenia are the pivotal clinical manifestations. 

Skeletal involvement, another important clinical feature of GD, is the most painful and debilitating symptom of the disease [2]. 

Nonetheless, the wide heterogeneity of onset symptoms of GD should always be firmly stressed.

In our patient the GD onset was particularly unusual because the osteoarticular symptom was a the only clinical symptom; moreover the absence of both peripheral cytopenia and significant liver/spleen enlargement could have been a cause of misdiagnosing or diagnostic delay [3]. 

In summary, our patient's illness began as bone crisis. As known, bone crises are acute episodes of severe skeletal pain and fever accompanied by leucocytosis and elevated erythrocyte sedimentation rates. 

Pediatric diseases that occur with a bone crisis are rather unusual: among these, there are hemato-oncologic diseases (e.g., sickle cell disease, myelodysplastic syndromes) and GD. Although bone crises are common in GD, they rarely are the presenting and only symptom of the disease, as in our case. 

Many other diseases (trauma, orthopedic diseases, infections such as acute osteomyelitis or septic arthritis, rheumatologic, or neurologic diseases) may present with symptoms that can mimic a bone crisis, therefore a thorough differential diagnosis is requested, particularly in otherwise healthy children.

It is worth noting that the bone crisis is mainly characterized by the excruciating pain that does not respond to NSAIDs. 

Moreover, MRI evaluation may represent a further diagnostic tool. In particular, in our experience the findings of an increased T2-weighted signal and an inhomogeneous contrast enhancement at femoral head and neck were strictly suggestive for bone crisis. 

Recently the International Collaborative Gaucher Group reported that 63% of patients experience bone pain and 26% develop bone crisis.

Major skeletal abnormalities evident at radiographic examinations are Erlenmeyer flask deformity or bone marrow infiltration (nearly 60%), osteopenia (50%), and bone infarction or avascular necrosis (33%).

At the time of diagnosis, children with GD type I show a high prevalence of bone disease (radiological bone disease is reported in 81% of cases, bone pain in 27% and bone crisis in 9%), probably still underestimated because bone disease is rarely evident on physical examination and may not correlate with the degree of visceral and haematological involvement [4].

Bone marrow infiltration may be responsible for osteonecrosis and affects predominantly femoral head, proximal humerus, and vertebral bodies leading to fractures and joint collapse. 

Osteopenia and cortical thinning is likely related to increased osteoclast activation by delivery of proinflammatory cytokine from storage cells which infiltrate bone.

Finally bone deformities of the distal femur and proximal tibia are due to defect in remodelling. The pathophysiology of inflammatory bone disease in GD remains poorly understood. Indeed, it is thought that the bone marrow infiltration by Gaucher cells, that is, lipid-engorged machrophages, may cause a localized inflammation leading to infarction and osteonecrosis, osteosclerosis, and osteolysis [5]. 

Moreover it is known that in Gaucher disease there are signs of cytokine-mediated systemic inflammation and an imbalance between osteoclast/osteoblast activities: notably increased plasma levels of interleukin-6 have been documented in GD patients [6]. 

In conclusion, our experience shows that GD may present in atypical way and simulate a relatively common condition in children such as acute hip joint pain. 

Moreover, the early recognition of GD can start safe and effective treatment with enzyme replacement which can decrease morbidity and reduce as far as possible the visceral and skeletal involvement.
